# Perioperative characteristics, histologic diagnosis, complications, and outcomes of dogs undergoing percutaneous drainage, sclerotherapy or surgical management of intrarenal cystic lesions: 18 dogs (2004–2021)

**DOI:** 10.1186/s12917-022-03327-z

**Published:** 2022-06-20

**Authors:** Michail Vagias, Guillaume Chanoit, Loretta J. Bubenik-Angapen, Erin A. Gibson, Hilde de Rooster, Ameet Singh, Valery F. Scharf, Janet A. Grimes, Mandy L. Wallace, Anne Kummeling, James A. Flanders, Georgios Evangelou, Ronan A. Mullins

**Affiliations:** 1 Department of Small Animal Surgery, Section of Small Animal Clinical Studies, University College Dublin, Belfield, Dublin 4, Ireland; 2 Langford Vets, University of Bristol, Langford, UK; 3 Sugar Land Veterinary Specialists, 1515 Lake Pointe Parkway, Sugar Land, TX 77478 USA; 4 Department of Surgical and Radiological Science, University of California-Davis School of Veterinary Medicine, Davis, CA USA; 5 Small Animal Department, Faculty of Veterinary Medicine, Ghent University, Merelbeke, Belgium; 6 Department of Clinical Studies, Ontario Veterinary College, University of Guelph, Guelph, Ontario Canada; 7 Department of Clinical Sciences, College of Veterinary Medicine, North Carolina State University, Raleigh, NC 27607 USA; 8 Department of Small Animal Medicine and Surgery, College of Veterinary Medicine, University of Georgia, Athens, GA 30602 USA; 9 Department of Clinical Sciences, Faculty of Veterinary Medicine, Utrecht University, Utrecht, The Netherlands; 10 Department of Clinical Sciences, College of Veterinary Medicine, Cornell University, Ithaca, NY 14850 USA; 11 AnimalCare Veterinary Center, 30 D-E, Glyfadas, Strovolos, 2023 Nicosia, Cyprus

**Keywords:** Intrarenal cystic lesion, Renal cyst, Dog, Deroofing, Sclerotherapy, Cyst drainage

## Abstract

**Background:**

Canine intrarenal cystic lesions (ICLs) are infrequently reported in the veterinary literature. Several treatment options have been described including cyst fenestration (partial nephrectomy/deroofing) +/− omentalization, sclerotherapy using alcohol as a sclerosing agent, percutaneous cyst drainage (PCD), and ureteronephrectomy. Information regarding presenting clinical signs, physical examination findings, histologic diagnosis and outcomes of dogs with ICLs treated by different methods is limited. Medical records of 11 institutions were retrospectively reviewed to identify dogs that underwent PCD, sclerotherapy, surgical deroofing +/− omentalization, or ureteronephrectomy for management of ICLs from 2004 to 2021. Six weeks postoperative/post-procedural follow-up was required. Cases suspected to represent malignancy on preoperative imaging were excluded. The study objective was to provide information regarding perioperative characteristics, complications, and outcomes of dogs undergoing treatment of ICLs.

**Results:**

Eighteen dogs were included, with 24 ICLs treated. Ten had bilateral. There were 15 males and 3 females, with crossbreeds predominating. PCD, sclerotherapy, deroofing and ureteronephrectomy were performed in 5 (5 ICLs treated), 7 (11 ICLs), 6 (6), and 7 (7) dogs, respectively, with 5 dogs undergoing > 1 treatment. Seven dogs experienced 8 complications, with requirement for additional intervention commonest. PCD, sclerotherapy and deroofing resulted in ICL resolution in 0/5, 3/11 and 3/6 treated ICLs, respectively. Histopathology identified renal cysts (RCs) in 7/13 dogs with histopathology available and neoplasia in 6/13 (4 malignant, 2 benign). Of 5 dogs diagnosed histopathologically with neoplasia, cytology of cystic fluid failed to identify neoplastic cells. Among 7 dogs with histologically confirmed RCs, 4 had concurrent ICLs in ipsilateral/contralateral kidney, compared with 2/6 dogs with histologically confirmed neoplasia.

**Conclusions:**

Benign and neoplastic ICLs were approximately equally common and cystic fluid cytology failed to differentiate the 2. Among renal-sparing treatments, deroofing most commonly resulted in ICL resolution. Presence of concurrent ICLs in ipsilateral/contralateral kidney does not appear reliable in differentiating benign from malignant ICLs.

## Background

Renal cysts (RCs) are infrequently reported in dogs and cats and are defined as intraparenchymal epithelium-lined cavities filled with liquid of various composition within the renal cortex or medulla [1, 2]. Current literature involving RCs in dogs and cats is limited to only 6 case reports [1, 3–7] and 1 retrospective case series [2]. Renal cystic lesions in dogs and cats are more frequently associated with polycystic kidney disease (PKD) or perinephric pseudocysts (PNPs) [4, 8–15]. Polycystic kidney disease is a hereditary condition most commonly affecting Persian or Persian-related cats [16, 17] but has also been described in Bull terriers, Cairn terriers and West Highland white terriers [8–13]. Cystic lesions associated with PKD have similar microscopic characteristics to RCs, however, those associated with the former are usually multiple, variably-sized and randomly distributed within the renal parenchyma of both kidneys and are not treated surgically [13, 17–19]. Conversely, PNPs, which are more commonly observed in cats, are fibrous fluid-filled cavities within the renal subcapsular or retroperitoneal space and lack an epithelial lining [14, 15, 20–26]. Only 2 cases of PNPs have been reported in dogs [4, 27].

Another less frequently described cystic lesion is the paraureteral pseudocyst (PUP), which has been observed adjacent to the kidney of dogs and cats [28–31]. This condition has also been referred to as urinoma and is defined as a retroperitoneal accumulation of extravasated urine confined within a fibrous sac [30].

In human medicine, renal cystic disease is a more heterogeneous entity that includes heritable, developmental and acquired disorders [32, 33]. Human renal cystic diseases have been classified as localized cystic disease (e.g. RCs), PKD, RCs observed with hereditary malformation syndromes (e.g. tuberous sclerosis, von Hippel-Lindau disease), glomerulocystic kidney disease, acquired renal cystic disease, renal medullary cysts, renal cystic dysplasia, extraparenchymal RCs (e.g. PNPs) and renal cell carcinoma with cystic changes [32, 33]. Renal cysts are common in people, with a prevalence of almost 10% in the general population [34, 35].

The etiopathogenesis of RCs in animals is currently unknown [5]. There is no known heritable nature to intraparenchymal RCs in dogs. Renal cysts can be incidental findings in clinically healthy dogs [1, 7], secondary to chronic nephropathies [2], or congenital in origin [4]. In human literature, RCs are mainly acquired lesions of unknown etiology [34]. It is believed that they may originate from weakening of the tubular basement membrane of the distal convoluted tubule or collecting duct cells, resulting in a diverticulum. This diverticulum may subsequently develop into a RC [36]. Reported risk factors for the development of RCs in humans include older age, male gender, smoking, systemic hypertension, and renal dysfunction [34–36]. No known risk factors have been identified for the development of RCs in animals.

Abdominal ultrasonography is the most commonly described imaging modality for the diagnosis of RCs in dogs [1, 3–5, 7]. The most common finding includes a solitary anechoic cystic structure within the renal parenchyma, with a thin, slightly hyperechoic, well-defined wall, and distant acoustic enhancement [1, 3–5, 7]. Reported RCs are usually unilateral and described as arising from different locations within the renal parenchyma affecting either the cranial or caudal pole of the kidney [1, 3–7].

Several treatment options for RCs have been described in veterinary literature with sclerotherapy using alcohol as a sclerosing agent being the most commonly performed treatment option [2, 3, 6, 7]. Less frequently described treatment options include cyst fenestration (partial nephrectomy/deroofing) and omentalization, percutaneous cyst drainage (PCD), and ureteronephrectomy [1, 4–6, 37].

Information regarding presenting clinical signs, physical examination findings, and outcomes of dogs with RCs treated by different methods is limited to 6 case reports [1, 3–7] and 1 retrospective case series containing only 5 dogs [2]. In addition, the postprocedural follow-up time of the dogs included in the case series is limited to 4 weeks. Furthermore, results of histopathologic analysis of RCs have not been routinely reported in the veterinary literature, with only 3 published cases describing histopathologically confirmed RCs [1, 4, 37]. Therefore, the objectives of this multi-institutional retrospective study were to report the perioperative characteristics, histologic diagnosis, complications, and outcomes of dogs undergoing PCD, sclerotherapy or surgical management of intrarenal cystic lesions (ICLs). In this study, the term ICL is used instead of RC as the term “cyst” is specific and indicates a benign histologic diagnosis.

## Results

### Signalment at presentation

Eighteen dogs were included in the study. Details regarding 1 of these dogs have been reported previously [38]. Breeds included mixed breed (*n* = 5); Yorkshire terrier (2); and 1 each of rat terrier, giant poodle, bichon frisé, miniature pinscher, Spanish water dog, English bulldog, Pembroke Welsh corgi, walker coonhound, Bouvier des Flandres, springer spaniel and Shih Tzu. There were 14 neutered males, 1 sexually intact male, 2 spayed females and 1 sexually intact female. Mean (SD) age was 10.6 (2.2) years, with 11 dogs older than 10 years. Mean (SD) weight was 19.2 (12.2) kg.

### Reason for presentation to contributing institution

Twelve of 18 dogs (66.7%) included in this study demonstrated clinical signs that may or may not have been attributable to renal cystic disease. These included decreased appetite (*n* = 3); lethargy (3); vomiting (2); polyuria/polydipsia (2); hematuria (2); abdominal distention (2); and 1 each of exercise intolerance, straining to defecate, whining, anorexia, collapse, abdominal pain, shaking, tense abdomen, panting, and weight loss. Five of 18 (27.8%) dogs did not demonstrate clinical signs at presentation that could be attributed to renal cystic disease, with the ICL being an incidental finding during diagnostic workup for a different condition. In 1 additional dog with suspected pancreatitis following chocolate ingestion, an ICL was incidentally identified on abdominal ultrasound.

### Concurrent/historical comorbidities

Concurrent/historical comorbidities were recorded in 12 of 18 (66.7%) dogs and included hypothyroidism (*n* = 4); hepatic mass/hepatomegaly (3); laryngeal paralysis (2); pancreatitis (2); osteoarthritis (2); prostatomegaly (2); rectal mass (2); testicular mass/enlargement (2); and 1 each of pancreatic cyst, intervertebral disc disease, chronic kidney disease, acute vestibular syndrome, intermittently painful thoracic limb, skin allergy, tremors, otitis externa, perineal hernia, perineal mass, probable tracheal collapse, hearing loss, and retinal degeneration.

### Abnormal physical examination findings at contributing institution

Abnormal physical examination findings were recorded in 14 of 18 (77.8%) dogs, 10 (71.4%) of which had abnormal findings deemed related to ICLs, which included a palpable abdominal mass (*n* = 5), tense abdomen (5), abdominal distention (4), pain on abdominal palpation (2), and a positive fluid wave (1). One dog that had undergone recent exploratory celiotomy by the referring veterinarian to investigate right renomegaly identified on abdominal radiographs was tense on abdominal palpation and demonstrated pain on palpation of its surgical wound.

### Blood pressure measurement at contributing institution

Blood pressure measurement was obtained on presentation to the contributing institution in 8 of 18 (44.4%) dogs. Blood pressure measurement was obtained by non-invasive methods in all dogs, however, the specific method was recorded in only 3 dogs and included oscillometric in 2 dogs and Doppler in 1 dog. Results of blood pressure measurement were available in 7 dogs and recorded as normotensive in 1 dog. Mean (SD) systolic blood pressure was 170.3 (18.7) mmHg, with systolic blood pressure greater than 140 mmHg in all 7 dogs [39].

### Results of clinicopathologic tests performed at presentation by either the referring veterinarian or at the contributing institution

Hematology was performed in 15 of 18 (83.3%) dogs, with abnormalities identified in 6 of 15 (40%) dogs, which included anemia, erythrocytosis, leukopenia, leukocytosis, thrombocytopenia, and thrombocytosis, alone or in combination.

Serum biochemical analysis was performed in 16 of 18 (88.9%) dogs. In the remaining 2 dogs, serum biochemical analysis was limited to renal values only. Abnormalities were observed in 13 of 18 (72.2%) dogs and included increases in blood urea nitrogen (*n* = 5), alkaline phosphatase (5), alanine aminotransferase (4), creatinine (3), and cholesterol (2); hypoalbuminemia (3), and hypoproteinemia (2). Other biochemical abnormalities included 1 each of hyperglobulinemia, hyperbilirubinemia, hyperglycemia, and increases in aspartate aminotransferase, gamma-glutamyltransferase, lipase, creatine kinase, and blood urea nitrogen/creatinine ratio.

Serum electrolyte concentrations were obtained in 14 of 18 (77.8%) dogs. Abnormalities were identified in 5 dogs and included hyperkalemia (*n* = 2); total hypercalcemia (2); and 1 each of hypokalemia, hypochloremia, hypocalcemia, hypomagnesemia, hypoferremia, hypophosphatemia, and increased sodium/potassium ratio.

Urinalysis was performed in 14 of 18 (77.8%) dogs. Method of urine collection was available for 9 dogs and included cystocentesis (*n* = 7) and free catch (2). Abnormalities were identified in 10 dogs and included proteinuria (*n* = 8); hematuria (6); pyuria (2); and 1 each of bilirubinuria, presence of casts, and presence of squamous epithelial cells. pH measurement was available in 12 dogs and was between 5.5–7.5 in 11 dogs and < 5.5 (acidic) in 1 dog [40]. Urine specific gravity measurement was available in 12 dogs, with values 1.015–1.050 (*n* = 9), > 1.050 (2) and 1.014 (1) [40].

Urine culture was performed in 8 of 18 (44.4%) dogs and was positive in 1 dog in which *Enterococcus faecalis* was isolated, the remaining dogs had no bacterial growth. Method of urine sample collection was via cystocentesis (*n* = 6) and not available (2). Two dogs had been receiving antibiotics at time of urine collection and both had negative urine cultures. For the remaining 6 dogs that had urine culture performed, information regarding whether they had received previous antibiotic medication was not available.

### Diagnostic imaging performed by either the referring veterinarian or at the contributing institution

#### Abdominal imaging

**Fig. 1:**
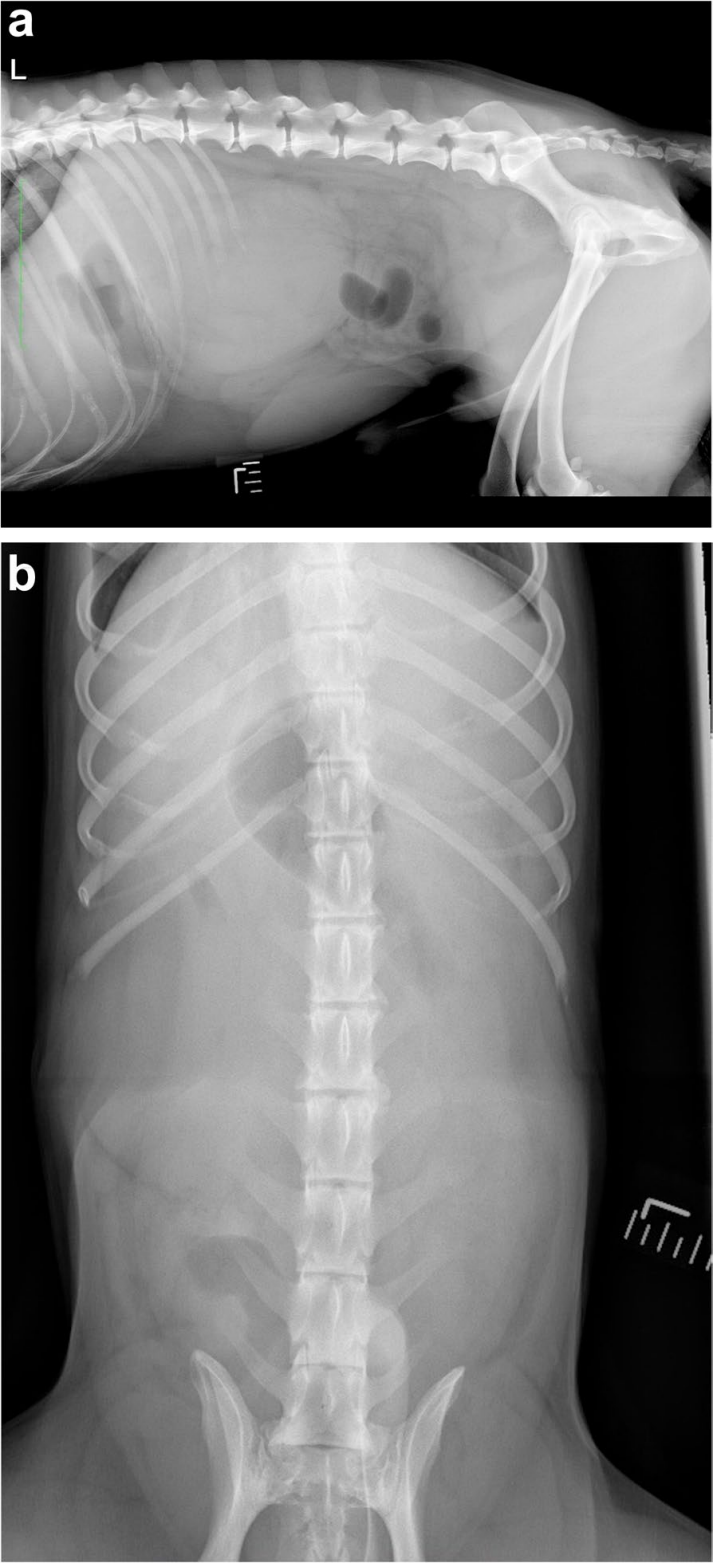
Lateral (**a**) and ventrodorsal (**b**) abdominal radiographs of a 5.2-year-old, 16.5 kg, neutered male, mixed breed dog treated with deroofing and diagnosed with renal cyst. A large ovoid soft tissue opacity is identified within the right craniodorsal abdomen, which causes a significant mass effect on surrounding abdominal structures. Images courtesy of Dr. Mullins

Radiographs of the abdomen were obtained in 10 of 18 (55.6%) dogs. Abnormal findings were identified in all 10 dogs and included an intra-abdominal mass (*n* = 7); unilateral renomegaly (2); prostatomegaly (2); and 1 each of bilateral renomegaly, cystic calculus, suspected right nephrolith, bilaterally misshaped kidneys, and hepatomegaly. The location of the intra-abdominal mass was within the cranial abdomen (*n* = 3), retroperitoneal space (2), or unspecified (2) (Fig. 1).

**Fig. 2:**
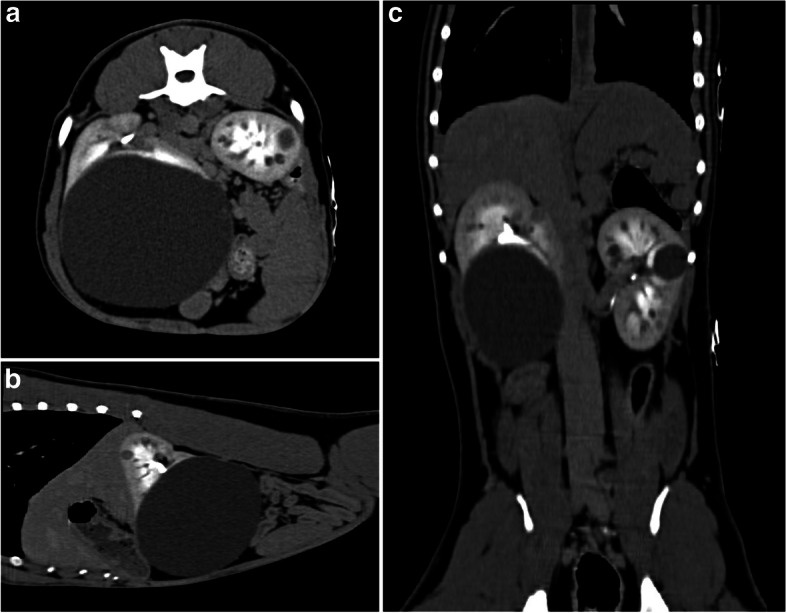
Transverse (**a**), sagittal (**b**) and dorsal (**c**) plain computed tomographic multiplanar reconstruction images of the same dog diagnosed with renal cyst in Fig. 1. Multiple, non-contrast enhancing, well-defined, ovoid structures of variable size and of fluid attenuation are visible within the cortex and medulla of both kidneys. The caudal pole of the right kidney contains a very large, ovoid, thin-walled, non-contrasting enhancing structure of fluid attenuation that has replaced the normal architecture of its caudal third. For figures (**a**) and (**c**), the left side of the dog is at the right side of the images. Images courtesy of Dr. Mullins

Abdominal ultrasound was performed in 17 of 18 (94.4%) dogs and abnormalities were identified in all 17. Contrast CT of the abdomen was performed in 7 of 18 (38.9%) dogs, with abnormalities identified in all 7. In 1 dog, the ICL appeared poorly contrast-enhancing but in the remaining 6 dogs no contrast uptake occurred (Fig. 2). Intrarenal cystic lesions affected only the left kidney in 8 dogs and both kidneys in 10 dogs at presentation. The median (range) size of the largest ICL at presentation on abdominal ultrasound or CT (whichever was larger if both performed) was 70 (32–240) mm.

**Fig. 3:**
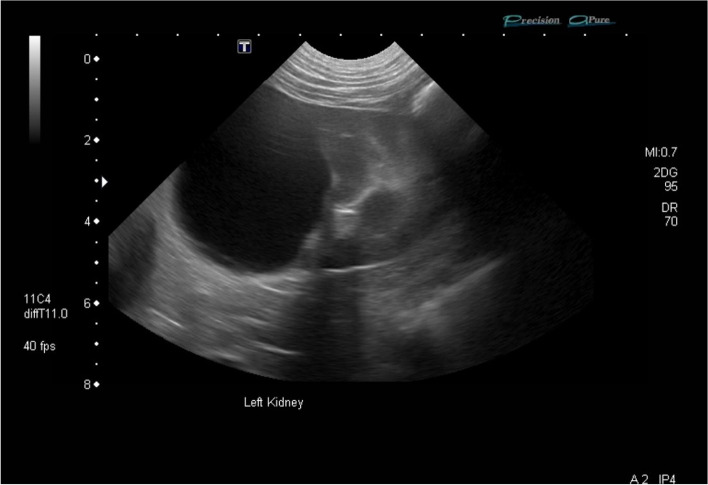
Ultrasonographic long-axis image of the left kidney of a 8.8-year-old, 41.9 kg, neutered male, mixed breed dog diagnosed with ICL treated with sclerotherapy (histopathology not available). A large anechoic cystic structure is identified within the caudal pole of the left kidney effacing the middle third of the renal parenchyma, has rounded turgid walls and lacks septations. Cranial is to the right of the image. Image courtesy of Dr. Grimes

The largest ICL affecting 1 or both kidneys was located at the cranial pole (*n* = 9), caudal pole (6), or middle third (4), with the location not specified in 9 dogs. In 1 dog, the ICL had ruptured resulting in free peritoneal fluid. The echogenicity of the contents of the ICL was described as anechoic (*n* = 5), particulate echoic (2), and 1 each of hypoechoic and mixed echogenicity (Fig. 3). The echogenicity of 10 ICLs was not specified. Pyelectasia was identified on abdominal ultrasound or CT in 8 of 18 (44.4%) dogs. Loss of corticomedullary definition of the affected kidney was identified in 5 of 18 (27.8%) dogs. Septations within the ICL were described on abdominal imaging in 3 of 18 (16.7%) dogs.

Only 1 of 8 dogs with unilateral ICL had additional cystic lesions identified within the same kidney. Of those with bilateral ICLs (*n* = 10), 7 had more than 1 ICL identified in the left kidney and 8 had more than 1 ICL identified in the right kidney at the time of presentation.

#### Thoracic imaging

Thoracic imaging was obtained in 10 of 18 (55.6%) dogs, including thoracic radiographs (*n* = 7) and computed tomography (4), with 1 dog receiving both. No significant abnormalities or evidence of metastatic disease was identified in any dog.

### Treatment

Of the 10 bilaterally affected dogs, the right kidney was treated in 4 dogs, the left kidney in 3 dogs, and both kidneys in 3 dogs.

#### Percutaneous cyst drainage

**Fig. 4:**
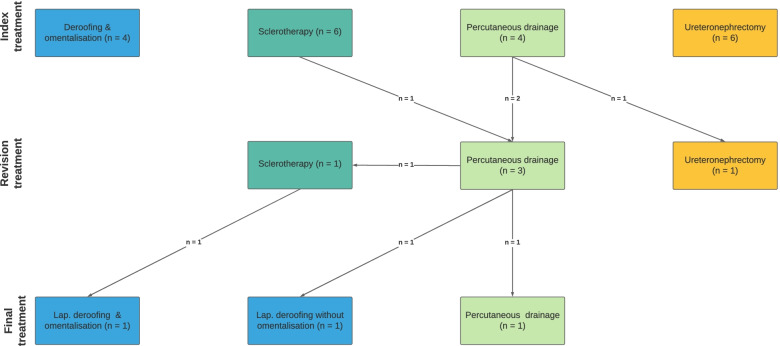
Flow diagram summarizing the different treatment procedures each dog underwent. Index treatment is defined as the first treatment performed

Percutaneous cyst drainage was performed as definitive treatment in 5 of 18 (27.8%) dogs, with 4 of 5 being performed as index treatment and 1 of 5 as revision treatment because of ICL recurrence 289 days after index sclerotherapy (Fig. 4). All 5 dogs that underwent PCD had only 1 ICL treated. Percutaneous cyst drainage was performed 3 times in 1 dog, twice in 1 dog, and once in 3 dogs (Table 1). Of the 8 PCD procedures performed in these 5 dogs, 2 were performed by the referring veterinarian and 6 at the contributing institution. No intraprocedural complications were recorded in 3 dogs that underwent PCD and this information was not available in 2 dogs. Postoperative complications were recorded in 4 of 5 (80%) dogs, all of which were major (requirement for additional intervention in 4 dogs, 1 of which developed hemoabdomen immediately after PCD resulting in euthanasia) (Table 1).

**Table 1:** Outcome of 5 dogs that underwent PCD

Dog number	Histologic diagnosis	Previous treatment	Clinical signs related to ICL at FU^a^	Resolution of preprocedural clinical signs at last FU^a^	Progression in size of treated ICL(s) at last FU imaging^a^	Azotemia pre-procedure	Azotemia on FU biochemistry^a^	Additional treatment performed	Dead, alive, LTFU post treatment
5	Cystadenocarcinoma	Sclerotherapy (289 days)	Yes, PU/PD, vomiting, hyporexia (236 days)	N/A	NP	NP	Yes (238 days)	Laparoscopic deroofing (241 days)	Died, unknown cause (443 days; 732 days post index treatment)
6	Transitional cell carcinoma with cyst formation	NP	Not available	N/A	92.5% its original size (14 days)	No	NP	Ureteronephrectomy (29 days)	Euthanized, progressive renal dysfunction (1245 days post index treatment)
9	NP	NP	No (42 days)	N/A	108.1% its original size (42 days)	No	NP	No	LTFU (42 days post index treatment)
10	Renal adenoma	NP	Not available	N/A	6 cm (original size not available) (8 days after second PCD)	No	No	Yes, repeat PCD (39 days); sclerotherapy (57 days); laparoscopic deroofing and omentalization (81 days)	Alive (406 days post index treatment)
16	NP	NP	Not available	N/A	100% its original size (78 days following second PCD)	Yes	Yes, progressively deteriorating (71 & 78 days after first & second PCD)	Yes PCD repeated twice (71 &149 days)	Euthanized due to hemoabdomen following 3rd PCD (Euthanized same day of 3rd PCD; 149 days post index treatment)

Days presented in parentheses are calculated from the date of the PCD

*FU* follow-up, *LTFU* lost to follow-up, *PU/PD* polyuria/polydipsia, *N/A* not applicable, *NP* not performed

^a^ Denotes date of last follow-up either after PCD or until the date of revision treatment

#### Sclerotherapy

**Fig. 5:**
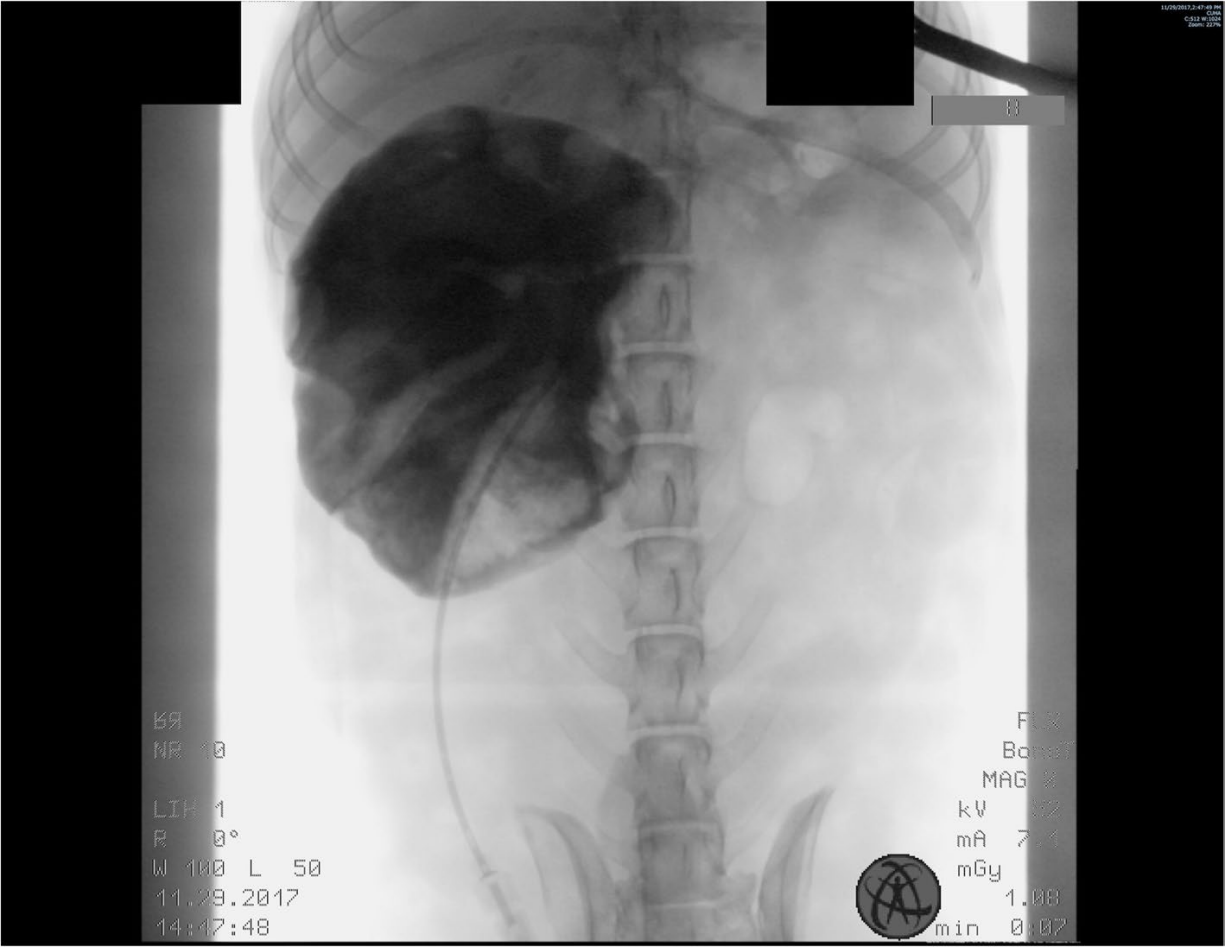
Ventrodorsal fluoroscopic image of a 14-year-old, 6.4 kg, neutered male, miniature pinscher diagnosed with renal cystadenocarcinoma of the right kidney. Intravenous contrast was injected percutaneously through a pigtail catheter to confirm its location and that the entirety of contrast remains within the ICL with no extracapsular leakage. Image courtesy of Dr. Flanders

Sclerotherapy was used to treat 11 ICLs in 7 of 18 (38.9%) dogs, with 6 of 7 as an index treatment and 1 of 7 as a revision treatment following 2 previous PCDs performed by the referring veterinarian prior to referral (Fig. 4). Sclerotherapy was performed only once in all 7 dogs at the contributing institution. Number of ICLs treated was 1 in 4 dogs, 2 in 2 dogs, and 3 in 1 dog. Sclerotherapy was performed percutaneously in 6 dogs and via open celiotomy in 1 dog. In 1 dog, intravenous contrast was injected percutaneously through a pigtail catheter to confirm its location within the ICL using fluoroscopy (Fig. 5). The sclerosing agent used included 95% ethanol (*n* = 5), ethanol of unknown concentration (1), and a 1:10 mixture of 2% lidocaine and 95% ethanol (1). Duration of sclerotherapy was 6 minutes in 2 dogs that received 2 injections of sclerosing agent, each lasting 3 minutes within the ICL, on a single occasion. In 2 dogs, the duration of sclerotherapy was 20 minutes, during which time the dog’s position was not changed. In the dog that underwent sclerotherapy by open celiotomy, the kidneys were rotated into different positions following instillation of the sclerosing agent. Duration of sclerotherapy was 40 minutes in 3 dogs, during which time each dog position was changed every 10 minutes from dorsal to right lateral, left lateral and ventral recumbency. Volume of sclerosing agent used expressed as a percentage of the volume of cystic fluid drained prior to sclerotherapy was 50% (*n* = 2); and 1 each of 2.5%, 19.6%, 22.2%, 26.9%, 48.0%, 55.6%, 66.7%, and 80%. This information was not available in 1 dog. An intraprocedural complication was recorded only in 1 dog which was categorized as minor. In this dog, perinephric sclerosing agent leakage with turbulence was identified during instillation of ethanol into the ICL, however, no intervention was required. Postprocedural complications were recorded in 2 dogs, both of which were major, and included requirement for additional intervention due to ICL recurrence (Table 2).

**Table 2:** Outcomes of 7 dogs that underwent sclerotherapy

Dog number	Histologic diagnosis	Previous treatment	Clinical signs related to ICL at FU^a^	Resolution of preprocedural clinical signs at last FU^a^	Progression in size of treated ICL(s) at last FU imaging^a^	Azotemia pre-procedure	Azotemia on FU biochemistry^a^	Additional treatment performed	Dead, alive, LTFU post treatment
3	NP	NP	No (571 days)	Yes	Cyst right kidney: 46.1% its original size; cyst left kidney resolved (22 days)	No	No (549 days)	No	Alive (571 days post index treatment)
5	Cystadenocarcinoma	NP	No (97 days)	Yes	100% its original size (97 days)	No	NP	PCD (289 days); Laparoscopic deroofing (530 days)	Died, unknown cause (732 days post index treatment)
7	Renal cyst	NP	No (44 days)	N/A	3 cysts treated: 78% its original size; 146.7% its original size; resolved (44 days)	No	No (13 days)	No	LTFU (44 days post index treatment)
8	NP	NP	No (278 days)	Yes	Resolved (258 days)	Yes	No (266 days)	No	Euthanized, vestibular disease (date unknown)
9	NP	NP	No (42 days)	N/A	41.7% its original size (42 days)	No	NP	No	LTFU (42 days post index treatment)
10	Renal adenoma	PCD performed twice (57 days and 18 days)	Not available	Not available	125% its original size (13 days)	No	NP	Laparoscopic deroofing and omentalization (24 days)	Alive (349 days; 406 days post index treatment)
13	NP	NP	No (914 days)	Yes	2 cysts treated: 22.2% & 125% its original size (117 days)	Yes	Yes but improved (117 days)	No	Alive (914 days post index treatment)

Days presented in parenthesis are calculated from the date of the sclerotherapy

*FU* follow-up, *LTFU* lost to follow-up, *N/A* not applicable, *NP* not performed

^a^ Denotes date of last follow-up either after sclerotherapy or until the date of revision treatment

#### Deroofing

**Fig. 6:**
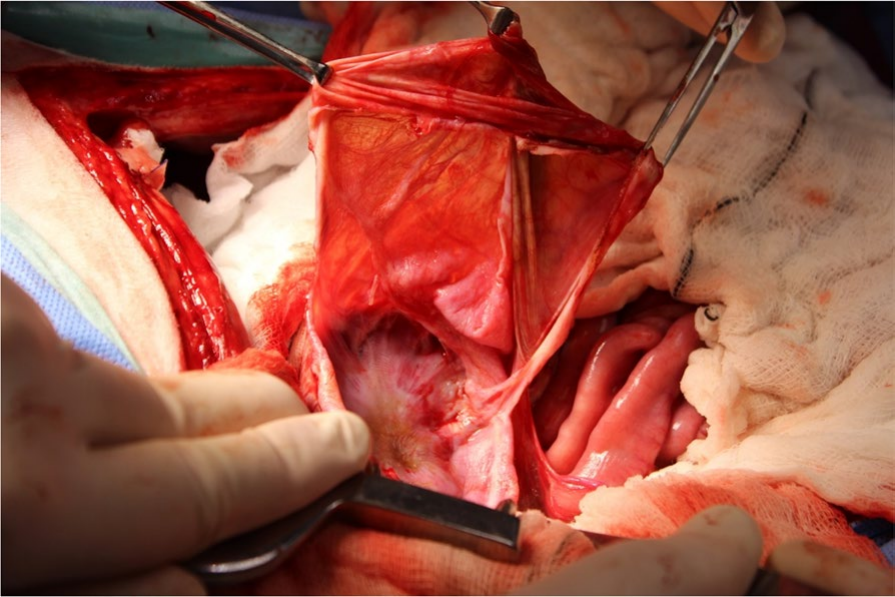
Intraoperative image during deroofing of the renal cyst of the same dog in Fig. 1. The wall of the renal cyst has been incised and its inner aspect is presented with the use of Allis tissue forceps prior to completion of deroofing. Cranial is to the top left of the image. Image courtesy of Dr. Mullins

**Fig. 7:**
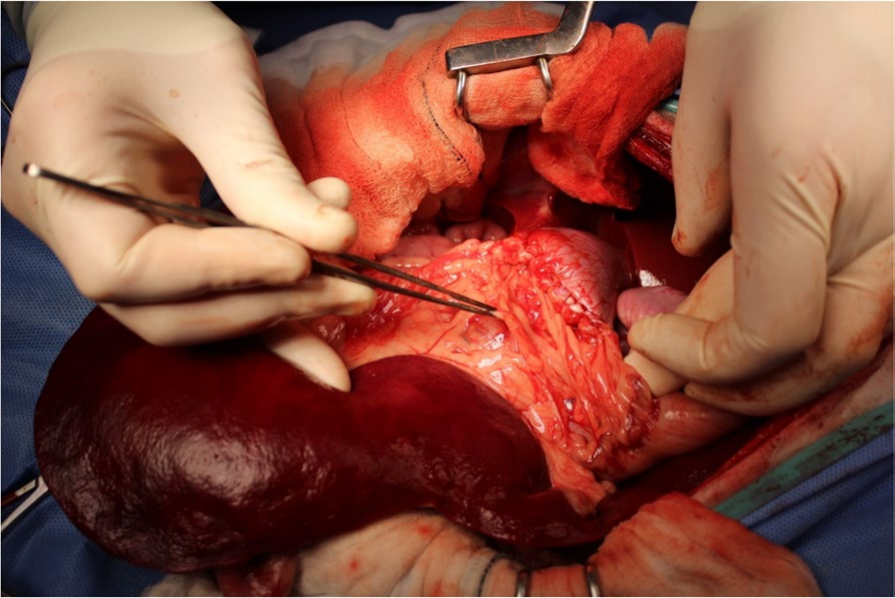
Intraoperative image during omentalization of the renal cyst of the same dog in Fig. 1 after deroofing. A double layer of a portion of the greater omentum in the region of the right kidney was sutured around the entire circumference of the remaining rim of cystic lining using absorbable monofilament suture in a simple continuous pattern. Cranial is to the right of the image. Image courtesy of Dr. Mullins

**Fig. 8:**
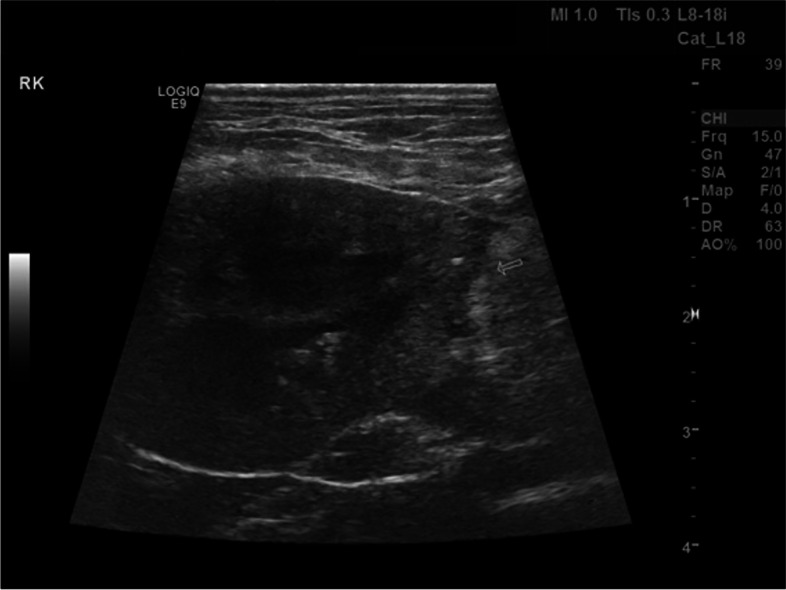
Long-axis ultrasonographic image of the right kidney of a 13-year-old, 5.6 kg, spayed female, Shih Tzu diagnosed with renal cyst of the right kidney. Image was obtained 6 weeks following deroofing and omentalization, and demonstrates the truncated appearance of the caudal pole of the kidney at the site of omentalization (arrow). Cranial is to the left of the image. Image courtesy of Dr. Mullins

**Fig. 9:**
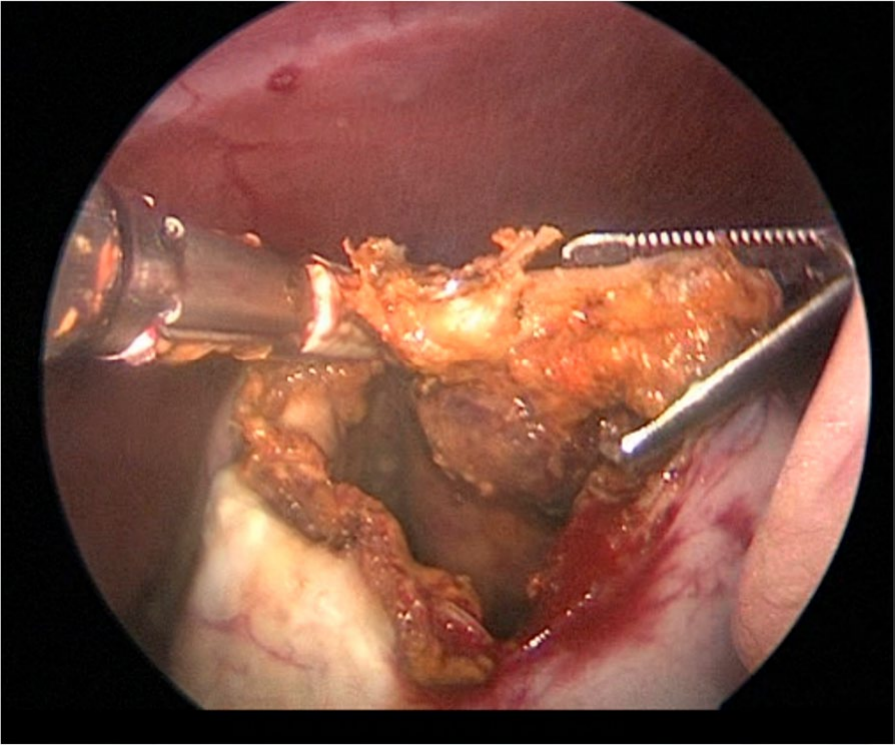
Intraoperative image during laparoscopic deroofing of the renal cystadenocarcinoma of the same dog in Fig. 5. The cystic wall was partially resected using bipolar vessel sealing device. Image courtesy of Dr. Flanders

Deroofing was performed in 6 of 18 (33.3%) dogs, with 4 of 6 as an index treatment and 2 of 6 as a revision treatment because of ICL recurrence following sclerotherapy (*n* = 1) and PCD (1) (Fig. 4). All 6 dogs had only 1 ICL treated with deroofing. Five of 6 (83.3%) dogs had deroofing performed with concurrent omentalization (Figs. 6, 7 & 8) and 2 (33.3%) had the surgery performed laparoscopically (1 with and 1 without omentalization), both as a revision treatment. In the 4 dogs that underwent open deroofing, the ICL wall was excised using a bipolar vessel sealing device (*n* = 2), monopolar electrosurgery (1), or unspecified technique (1). For 1 dog in which a bipolar vessel sealing device was used, the exposed intrarenal surface was lasered following deroofing and prior to omentalization. For both dogs that underwent laparoscopic deroofing, surgery was performed using a bipolar vessel sealing device (*n* = 1) or ultrasonic vessel sealing device (1) (Fig. 9). No intraoperative complications were recorded in any dog. One dog developed a minor postoperative complication, which included acute lethargy and vomiting 6 days after surgery that responded to medical management. No dog experienced a major postoperative complication.

#### Ureteronephrectomy

**Fig. 10:**
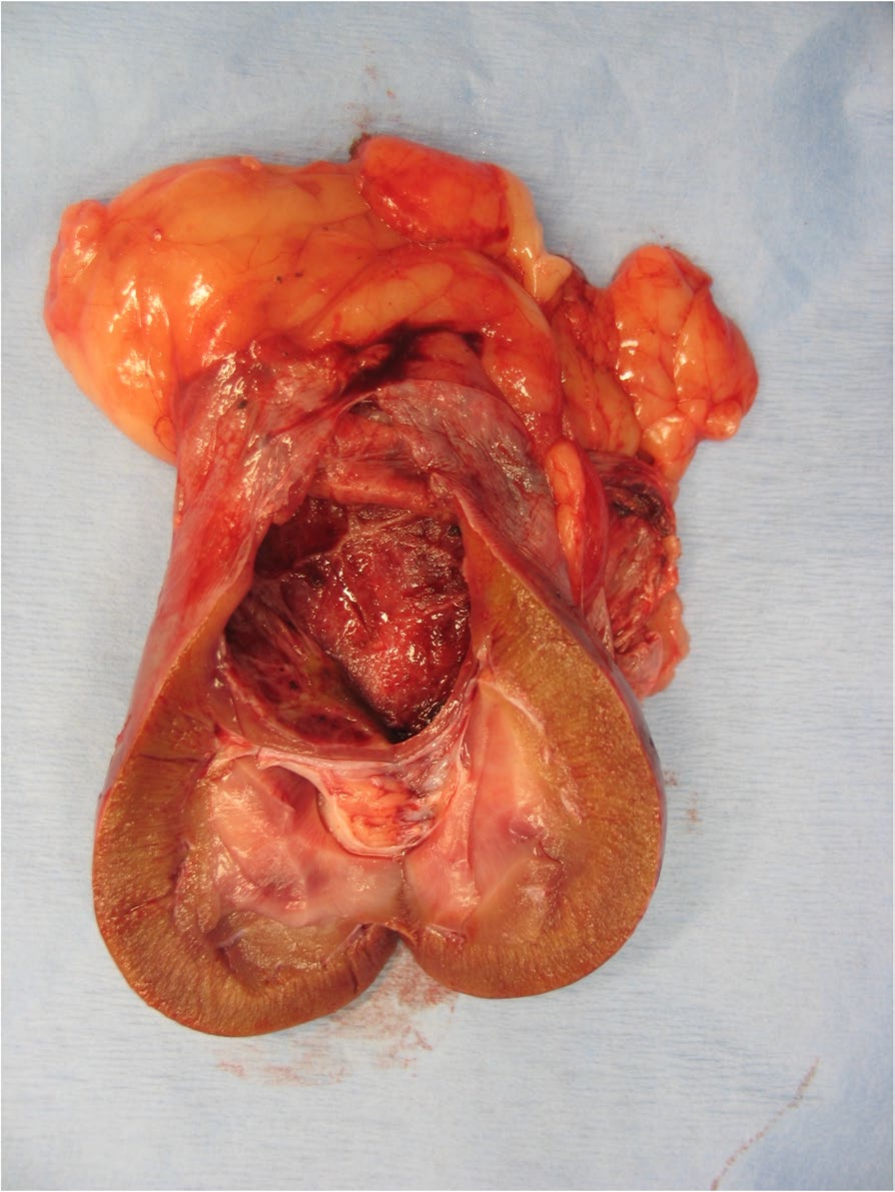
Macroscopic appearance of the excised left kidney (incised sagittally) of a 11.4-year-old, 29.1 kg, neutered male, giant poodle diagnosed with renal cyst. A thin-walled cyst is identified at caudal pole of left kidney replacing the normal parenchyma. Image courtesy of Dr. de Rooster

Ureteronephrectomy was performed in 7 of 18 (38.9%) dogs, with 6 of 7 as an index treatment and 1 of 7 as a revision treatment because of ICL recurrence following PCD (Fig. 4). Six of 7 dogs had only 1 ICL treated with ureteronephrectomy, and the remaining dog had a large ICL with additional multiple small cortical ICLs within the excised kidney (Fig. 10). No intraoperative complications were recorded. One minor postoperative complication was recorded consisting of aspiration pneumonia and pancreatitis that were successfully treated medically. No dog experienced a major postoperative complication.

Overall, 1 of 18 (5.6%) dogs experienced 1 intra-procedural/operative complication (perinephric fluid leakage during sclerotherapy), and 6 of 18 (33.3%) experienced 7 post-procedural/operative complications (requirement for additional intervention because of ICL recurrence [*n* = 4]; and 1 each of hemoabdomen following PCD, acute lethargy and vomiting following deroofing, and aspiration pneumonia and pancreatitis following ureteronephrectomy).

### Clinicopathologic and microbial testing of cystic fluid

Cytologic analysis of cystic fluid was performed in 11 of 18 (61.1%) dogs and was obtained preprocedurally with ultrasound guidance in 3 dogs, and intraprocedurally in 5 dogs. Fluid collection was not specified in 3 dogs. Gross appearance of the fluid was described in 5 of 11 dogs and included clear or cloudy/turbid, straw-yellow, hemorrhagic, or brown-colored fluid. A detailed cytologic report was available for 9 of 11 dogs; 1 dog had a non-diagnostic sample obtained and an additional dog had no detailed report available, however, cytologic findings were reported to be consistent with RC. Cellularity was described as low (*n* = 6), low-to-moderate (1), or not characterized (2). Erythrocytes were most commonly described (*n* = 7), followed by macrophages (4), degenerate or non-degenerate neutrophils (3), and renal epithelial cells (1), with cell debris described in 5 dogs. The background was described in 6 dogs and was proteinaceous in all cases. No bacteria were documented in any of the 9 detailed cytologic reports available.

For 2 dogs in which the ICL had ruptured preoperatively, cytologic analysis of free peritoneal fluid obtained either intraoperatively or by abdominocentesis was performed and identified as grossly dark or brown colored fluid. The fluid was described as exudate in 1 dog, with suppurative inflammation and evidence of previous hemorrhage, and unclassified in the other dog, with mostly cellular debris consisting of erythrocytes and neutrophils identified on microscopic examination. No bacteria were identified in either dog.

Nine of 13 dogs which had cytology of ICL or free peritoneal fluid performed also had histopathology performed, with neoplasia histopathologically diagnosed in 5 of 9 dogs. No cytologic evidence of neoplasia was identified in any dog.

Bacterial culture of the ICL (*n* = 8) or free peritoneal fluid (2) yielded no bacterial growth. Culture of a swab obtained from 1 ICL also yielded no growth.

Measurement of ICL creatinine concentration was performed in 4 dogs and was similar to serum creatinine in all cases, and therefore not consistent with urine.

### Histopathology

Histopathology was performed in 13 of 18 (72.2%) dogs, 6 after deroofing and 7 after ureteronephrectomy. Histopathology was consistent with a RC in 7 (53.8%) dogs, benign neoplasia in 2 (15.4%), and malignant neoplasia in 4 (30.8%) dogs. Benign neoplasms included papillary cystadenoma (*n* = 1), and renal adenoma with cyst formation (*n* = 1). Malignant neoplasms included 1 each of papillomatous cystadenocarcinoma, cystadenocarcinoma with granulomatous inflammation, transitional cell carcinoma with cystic component, and renal cell carcinoma with cystic component.

Of the 4 dogs that were diagnosed with malignancy, 2 were treated with ureteronephrectomy alone, 1 was treated with PCD followed by ureteronephrectomy because of ICL recurrence, and the remaining dog underwent initial sclerotherapy followed by PCD and later laparoscopic deroofing because of ICL recurrence (Fig. 4). Of the remaining 9 dogs that were diagnosed with benign neoplasms or RC, revision treatment was required in 1 dog, in which PCD was performed as the index treatment followed by sclerotherapy and laparoscopic deroofing with omentalization because of ICL recurrence.

Of 7 dogs with histologically confirmed RCs, 4 (57.1%) had more than 1 ICL in the ipsilateral or contralateral kidney at the time of presentation. Conversely, of 6 dogs with histologically confirmed neoplasms (benign and malignant), 2 (33.3%) had more than 1 ICL in the ipsilateral or contralateral kidney at the time of presentation.

### Outcome

#### Percutaneous cyst drainage

Outcomes of 5 dogs that underwent PCD are presented in Table 1. Histopathologic diagnosis was available in 3 of 5 (60%) dogs, all of which were diagnosed with neoplasia, and underwent eventual deroofing (*n* = 2) or ureteronephrectomy (1). Clinical signs related to ICL(s) at last follow-up examination performed (either after PCD or until the date of revision treatment) were present in only 1 dog, which included polyuria/polydipsia, vomiting and hyporexia. This dog did not have any clinical signs prior to PCD and had undergone previous sclerotherapy. In 3 dogs, information regarding clinical signs related to ICL(s) was unavailable. Resolution of preprocedural clinical signs following PCD could not be evaluated as ICL(s) were an incidental finding in 2 dogs, and information regarding clinical signs related to ICL(s) was unavailable in 3 dogs. Four of 5 dogs had follow-up imaging performed and progression in size of treated ICL(s) at last follow-up (after PCD or up until the date of revision treatment) was documented in 3 of 4 dogs, with ICLs > 50% of their original size before PCD. In the remaining dog, a 6 cm ICL was identified at the contributing institution but the original size of the ICL was not available as both previous PCD procedures were performed by the referring veterinarian. Follow-up biochemistry was available in 3 dogs, with azotemia (increase in urea or creatinine or both) identified in 2 dogs on last biochemistry performed. One of the dogs with azotemia was azotemic before PCD and worsening of the azotemia was observed after treatment. The other dog was not azotemic prior to previous sclerotherapy but repeat biochemistry was not performed immediately prior to PCD. Four of 5 (80%) dogs required revision treatment following PCD because of ICL recurrence. The dog that did not have revision treatment was found to have an ICL 108.1% its original size, 42 days after PCD and had no clinical signs at that stage.

#### Sclerotherapy

Outcomes of 7 dogs that underwent sclerotherapy are presented in Table 2**.** Of these 7 dogs, only 3 (42.9%) had histopathology performed, with neoplasia identified in 2 dogs and RC in 1. Neoplasia in the former 2 dogs consisted of renal adenoma (*n* = 1) and cystadenocarcinoma (*n* = 1). Information regarding presence of clinical signs related to ICL(s) at last follow-up examination performed (either after sclerotherapy or until the date of revision treatment) was available for 6 of 7 (85.7%) dogs, with absence of clinical signs in all 6 at a mean (SD) of 324.3 (352.3) days. Resolution of preprocedural clinical signs was observed in 4 of 5 dogs (ICLs were incidentally diagnosed in 2 additional dogs), with information regarding presence of postprocedural clinical signs not available for 1 dog. Of a total of 11 ICLs treated with sclerotherapy in 7 dogs, 3 ICLs were found to be involuted at last follow-up after sclerotherapy or until the date of revision treatment, 3 ICLs were found to be < 50% their original size, and 5 were found to be ≥50% their original size. Follow-up biochemistry was performed in 4 of 7 (57.1%) dogs, with azotemia identified (but improved) at last biochemistry in 1 dog with preprocedural azotemia. Two of 7 (28.6%) dogs treated with sclerotherapy underwent revision treatment, which included PCD after 289 days followed by laparoscopic deroofing at 530 days (*n* = 1) and laparoscopic deroofing with omentalization after 24 days (*n* = 1). Both were subsequently diagnosed with neoplastic ICLs (renal adenoma and cystadenocarcinoma).

#### Deroofing

Outcomes of 6 dogs that underwent deroofing are presented in Table 3. Histopathology was available for all 6 dogs, with RCs diagnosed in 4 dogs and neoplasia in 2 (cystadenocarcinoma and renal adenoma). Information regarding presence of clinical signs related to ICL(s) at last follow-up examination performed after deroofing was available for all dogs, with absence of clinical signs in 5 of 6 (83.3%) dogs at a mean (SD) of 268.2 (238.7) days. One dog had persistent hyporexia 202 days after laparoscopic deroofing. Resolution of preprocedural clinical signs was observed in 2 of 4 dogs (ICLs were incidentally diagnosed in 2 dogs), with this information not available for 1 dog. The dog with hyporexia after laparoscopic deroofing was also hyporexic prior to deroofing. The ICLs had resolved at the time of last abdominal imaging follow-up in 3 of 4 (75%) dogs diagnosed with RCs, and in the remaining dog the RC was 28% of its original size 44 days after deroofing. In the 2 dogs diagnosed with neoplasia, the ICL was found to have regained > 50% of its pre-deroofing size at 325 days postoperatively (*n* = 1) and was not assessed by follow-up imaging (1). Azotemia was present in 3 of 6 (50%) dogs on last follow-up biochemistry performed, all of which were azotemic before treatment (azotemia improving [*n* = 2] and worsening [1]). No dog underwent revision treatment.

**Table 3:** Outcomes of 6 dogs that underwent deroofing +/− omentalization

Dog number	Histologic diagnosis	Previous treatment	Clinical signs related to ICL at FU^a^	Resolution of preprocedural clinical signs at last FU^a^	Progression in size of treated ICL(s) at last FU imaging^a^	Azotemia pre-procedure	Azotemia on FU biochemistry^a^	Additional treatment performed	Dead, alive, LTFU post treatment
5	Cystadenocarcinoma	Sclerotherapy (530 days), PCD (241 days)	Decreased appetite (202 days)	No	NP	Yes	Yes but improved (18 days)	No	Died, unknown cause (202 days; 732 days post index treatment)
7	Renal cyst	NP	No (44 days)	N/A	28% its original size (44 days)	No	No (13 days)	No	LTFU (44 days post index treatment)
10	Renal adenoma	PCD performed twice (81 days and 42 days), Sclerotherapy (24 days)	No (325 days)	Not available	62.5% its original size (325 days)	NP	No (325 days)	No	Alive (325 days; 406 days post index treatment)
12	Renal cyst	NP	No (619 days)	Yes	Resolved (61 days)	No	No (61 days)	No	Alive (619 days post index treatment)
14	Renal cyst	NP	No (308 days)	N/A	Resolved (51 days)	Yes	Yes, worsening (51 days)	No	Alive (308 days post index treatment)
18	Renal cyst	NP	No (45 days)	Yes	Resolved (45 days)	Yes	Yes, improving (45 days)	No	Alive (45 days post index treatment)

Days presented in parenthesis are calculated from the date of the deroofing +/− omentalization

*FU* follow-up, *LTFU* lost to follow-up, *N/A* not applicable, *NP* not performed

^a^Denotes date of last follow-up after deroofing +/− omentalization

#### Ureteronephrectomy

Outcomes of 7 dogs that underwent ureteronephrectomy are presented in Table 4. Histopathology was available for all 7 dogs, with neoplasia diagnosed in 4 dogs and RC in 3 dogs. Neoplasia in 4 dogs consisted of papillomatous cystadenocarcinoma (*n* = 1), papillary cystadenoma (1), transitional cell carcinoma (1), and renal cell carcinoma (1). Information regarding presence of clinical signs related to ICL(s) at last follow-up examination performed after ureteronephrectomy was available for 6 of 7 (85.7%) dogs, with absence of clinical signs in 4 dogs at a mean (SD) of 262.5 (173.5) days. The remaining 2 dogs had decreased appetite at the time of last follow-up examination. Information regarding preprocedural clinical signs was available in 5 dogs and resolution was achieved in all 5 dogs. This information was not available for 2 dogs as no follow-up clinical examination was performed postoperatively (*n* = 1) or clinical signs were not available prior to ureteronephrectomy (1). No ICLs were found to have formed in the remaining kidney in 3 of 5 (60%) dogs that had follow-up imaging performed. In the other 2 dogs, ICLs were diagnosed in the remaining kidney (1 dog had newly formed ICLs and 1 had previously identified ICLs). In both dogs, the ICLs appeared static in size on repeat imaging performed. Follow-up biochemistry was performed in 5 of 7 (71.4%) dogs, with azotemia identified in 4 of 5 (80%) dogs. Azotemia persisted (but improved) postoperatively in 2 dogs that had azotemia prior to ureteronephrectomy, and developed postoperatively in the remaining 2 dogs that did not have azotemia prior to ureteronephrectomy. In 1 dog, azotemia did not occur before or after surgery. No dog underwent revision treatment following ureteronephrectomy.

**Table 4:** Outcomes of 7 dogs that underwent ureteronephrectomy

Dog number	Histologic diagnosis	Previous treatment	Clinical signs related to ICL at FU^a^	Resolution of preprocedural clinical signs at last FU^a^	ICL(s) in remaining kidney at last FU imaging^a^	Azotemia pre-procedure	Azotemia on FU biochemistry^a^	Additional treatment performed	Dead, alive, LTFU post procedure
1	Renal cyst	NP	NP	N/A	NP	No	NP	No	Euthanized due to nasal ADC (196 days post index treatment)
2	Papillomatous cystadenocarcinoma	NP	No (346 days)	Yes	No (120 days)	No	NP	No	LTFU (346 days post index treatment)
4	Papillary cystadenoma	NP	No (282 days)	Yes	NP	No	No (4 days)	No	Alive (282 days post index treatment)
6	Transitional cell carcinoma with cyst formation	PCD (29 days)	Yes, decreased appetite (1216 days)	Not available	Yes, 2 new cyst remaining static in size (1147 days)	No	Yes (1147 days)	No	Euthanized due to progressive renal dysfunction (1216 days; 1245 days post index treatment)
11	Renal cell carcinoma	NP	No (408 days)	Yes	No (106 days)	Yes	Yes but improved (106 days)	No	Alive (408 days post index treatment)
15	Renal cyst	NP	No (14 days)	Yes	No (361 days)	No	Yes (361 days)	No	Euthanized, multicentric lymphoma (1069 days post index treatment)
17	Renal cyst	NP	Yes decreased appetite (251 days)	Yes	Yes, static preoperatively diagnosed ICLs (102 days)	Yes	Yes but improved (102 days)	No	Died, unknown cause (251 days post index treatment)

Days presented in parenthesis are calculated from the date of the ureteronephrectomy

*FU* follow-up, *LTFU* lost to follow-up, *N/A* not applicable, *NP* not performed, *ADC* adenocarcinoma

^a^ Denotes date of last follow-up after ureteronephrectomy

### Survival

After index treatment, 8 of 18 (44.4%) dogs were alive after a mean (SD) of 444.1 (260.3) days, 7 of 18 (38.9%) were dead after a mean (SD) of 607.0 (477.8) days and 3 of 18 (16.7%) dogs were lost to follow-up after 42, 44 and 346 days, respectively. Death was related to renal disease in 3 of 7 (42.9%) dogs, non-renal disease-related causes in 2 of 7 (28.6%) dogs and unknown cause in 2 of 7 (28.6%) dogs.

## Discussion

In our study, ICLs were most commonly diagnosed in older dogs (mean age of 10.6 years). This finding is in agreement with previously published literature, with RCs most commonly identified in dogs 8–14 years of age [1, 3, 5–7], with reports of affected dogs less than 8 years of age being sparse [4]. In humans, the incidence of simple RCs (defined as those with good acoustic enhancement, absence of echoes within the lesion, and sharply marginated smooth walls) [41] increases with age, ranging from 0.22 to 0.55% in children and up to 36% in people over 80 years of age [34]. No breed predisposition was identified in our study with mixed breed dogs most commonly affected. Renal cysts have been described in a variety of breeds in the literature including American Staffordshire terrier, German shepherd, Yorkshire terrier, mixed breed dogs, and miniature pincher [1, 3, 5–7]. Males were overrepresented in our study, representing 15 of 18 (83.3%) affected dogs. In the veterinary literature, an equal distribution of male [1, 3, 7] and female [5, 6, 42] dogs has been reported. In humans with simple RCs, a male-to-female ratio of 2.8:1 in adults and 1.6:1 in children has been identified [35, 43]. The male predisposition identified in our study should be interpreted with caution due to the relatively low number of dogs included in the study. Interestingly, no sex predilection has been identified for other renal cystic diseases such as PKD and PNPs in dogs and cats [9, 10, 14, 16, 44–47].

In 6 of 18 dogs in our study, ICLs were an incidental finding, with 5 dogs having no clinical signs and 1 dog having clinical signs related to acute pancreatitis. In 3 previously published canine cases, the RC was an incidental finding during either a routine evaluation following previous mast cell tumor excision [7], further investigation for multiple mammary masses [1], or a regular health check [1]. In humans, RCs are mostly asymptomatic and incidentally identified [34]. Of the dogs that were symptomatic in our study, the most common presenting signs were decreased appetite and lethargy, which is similar to what has been previously described in veterinary literature [2, 5, 6]. In humans, < 10% of RCs become symptomatic, with symptoms including flank pain, flank mass, hypertension, hematuria, and fever secondary to infection [34, 36]. In 1 multi-institutional retrospective study involving children with symptomatic RCs, the most common reported symptom was abdominal pain [43].

Abnormalities during physical examination were commonly identified in our study, with a palpable abdominal mass, tense abdomen, and abdominal distention most frequent. Pain on abdominal palpation was a common finding in a previous retrospective case series involving dogs and cats but was only identified in 2 dogs in our study [2]. The identification of an enlarged and irregular kidney on abdominal palpation was a consistent finding in all 6 previously reported cats with RCs [2, 37]. While the presence of a palpable abdominal mass was a relatively common finding (5 of 18) in the dogs of our study, this appears uncommon based on previously published canine RC cases, reported in only 1 of 11 dogs [1–7].

Systemic hypertension has been described as a common finding in dogs and cats with RCs [2]. Systolic blood pressure measurements had values > 140 mmHg were identified in 7 dogs in which blood pressure was measured in our study. Similarly, in a previous retrospective study, systemic hypertension was identified in all 5 dogs with RCs (≥160 mmHg) [2]. In humans, it has been shown that the presence of simple RCs is associated with increased prevalence of systemic hypertension with older male patients being more commonly affected [48–50]. Furthermore, human patients with multiple RCs, large RCs or peripherally located RCs have a higher likelihood of being hypertensive [48–50].

In humans, a CT based classification system known as the Bosniak classification is routinely used for categorizing renal cystic lesions according to their likelihood of malignancy [51]. This classification includes 5 categories, namely Bosniak categories (BC) I, II, IIF, III and IV [51]. Studies correlating histopathology of cystic lesions and their classification according to Bosniak system identified that 0% of BC I lesions, 0.09% of BC II lesions, approximately 10% of BC IIF, approximately 50% of BC III lesions, and 90% of BC IV lesions were malignant [51–54]. In humans this classification is used to guide the most appropriate treatment, with BC I and II lesions typically ignored, BC IIF lesions monitored, and BC III and IV excised unless substantial comorbidities or limited life expectancy would favor observation instead [51]. In our study, the choice of treatment was based on surgeon preference. In line with the study inclusion and exclusion criteria, malignancy was not suspected in any of the cases herein on preoperative imaging or cytologic analysis and therefore did not influence the surgeon’s decision regarding the most appropriate treatment. A classification system similar to the Bosniak is lacking in veterinary medicine and may be useful to guide the most appropriate treatment in animals with ICLs. This is highlighted by the fact that 6 of 13 dogs with histopathology in our study were diagnosed with neoplasia, 4 of which were malignant.

Based on the results of our study, PCD was the least successful treatment, with 4 of 5 dogs undergoing additional treatment (repeat PCD or different) because of ICL recurrence. In our study, we included dogs in the PCD category only when ICL drainage was intended to be a definitive treatment and not just for the purpose of obtaining a sample for cytologic analysis. This technique has only been described in 1 other previous case, with the RC found to have regained almost 100% its original size after 1 month [6]. In humans, PCD of simple RCs is associated with a high recurrence rate of up to 80% and therefore is considered an ineffective treatment option [34, 36, 55, 56]. This high recurrence rate is related to continued fluid production associated with the presence of fluid-secreting epithelium lining the cyst wall [55].

Unlike PCD alone, sclerotherapy is aimed at destroying the secretory epithelium of RCs and is the most commonly described treatment for RCs in dogs and cats [2, 3, 6, 7, 55]. In people, no consensus has been reached regarding the most successful sclerotherapy protocol with regard to volume of sclerosing agent, duration of treatment, number of treatments, however, ethanol is the most commonly used sclerosing agent [34, 36]. Similarly, ethanol was used in all cases that underwent sclerotherapy in our study. Two different sclerotherapy protocols have been described for the management of RCs in veterinary literature and were also used in 4 of 7 dogs in our study. One protocol involved 2 injections on a single occasion of 95% ethanol followed by drainage 3 minutes after each injection [7]. The second protocol involved a single injection of 95% ethanol left in situ for 20 minutes during which time the position of the patient was changed every 5 minutes to ensure uniform distribution of the sclerosing agent [3]. A modification of the latter protocol was used in 3 of 7 dogs of the present study where the sclerosing agent was left intracavitary for 40 minutes in an effort to increase the chances of destruction of the secretory epithelium. In humans, prolonged contact of the secretory epithelium with the sclerosing agent has been shown to result in a lower rate of cyst recurrence [55, 57].

The overall rate of intra-procedural/operative complications in our study was low, with only a minor intraprocedural complication having occurred during sclerotherapy (identification of mild perinephric leakage of the sclerosing agent), which was not associated with patient morbidity and did not require intervention. Leakage of sclerosing agent is a rare complication in people that usually does not result in serious problems [36]. It is also possible that a certain degree of leakage is to be expected and that the occurrence of this complication in dogs is underreported. In a previous report [2], mild abdominal hemorrhage requiring no intervention was described as an intraoperative complication during sclerotherapy in 1 cat and 1 dog but was not identified in our study. No intraprocedural or postprocedural complications have been reported for canine RCs treated with PCD, ureteronephrectomy or cyst fenestration/omentalization [1, 4–6]. The highest rates of post-procedural/operative complications occurred after PCD and sclerotherapy in our study and included the requirement for revision treatment because of ICL recurrence in most cases. The rate of postoperative complications after deroofing and ureteronephrectomy was low and none required further intervention.

In our study, 3 of 11 ICLs treated with sclerotherapy were found to have involuted at last follow-up imaging (either after sclerotherapy or until the date of revision treatment), 3 were < 50% their original size and 5 were ≥ 50% their original size. Of the 8 ICLs that did not involute, only 2 underwent revision treatment, both of which were ≥ 50% their original size at follow-up imaging. Of the remaining 6 ICLs that did not involute, 3 were < 50% their original size and 3 were ≥ 50% their original size. These findings, however, should be interpreted with caution because whether these 8 ICLs would have eventually involuted with longer follow-up imaging is unknown. In humans, complete cyst regression can take in excess of 12 months, and therefore, persistence of the cyst on follow-up abdominal ultrasound prior to this time may not signify treatment failure [58]. In the early follow-up period, cyst refilling following sclerotherapy may be secondary to reactive or inflammatory fluid collections, which may eventually disappear [58]. A similar phenomenon has been described in 2 dogs in which the RC partially refilled following sclerotherapy before complete resolution at 1 and 8 months, respectively [3, 6].

Based on the inclusion/exclusion criteria of our study, we did not include ICLs that were suspected to be neoplastic on initial imaging performed. Interestingly, 6 of 13 ICLs with histopathology available were diagnosed with neoplasia, with 4 of these 6 being consistent with malignancy. Primary renal neoplasms are rare in dogs and cats, representing less < 2% of all tumors in these species, with renal cell carcinoma being the most common malignant tumor [59, 60]. Cystadenocarcinoma was found in 2 of 4 dogs with malignant neoplasia in our study. This neoplasm has been most frequently described in German shepherd dogs and infrequently in other breeds, is usually bilateral in presentation, and is associated with concurrent nodular dermatofibrosis [61, 62]. Both affected dogs in our study were toy breeds (bichon frisé and miniature pinscher), neither had skin nodules and only 1 had bilateral ICLs. All 6 dogs with neoplastic ICLs in our study had preoperative/intraoperative cytologic analysis of cystic fluid performed and no evidence of neoplastic cells were identified in any dog. In humans with renal cystic masses, cytology is not commonly performed due to its low sensitivity (50%) in detecting malignancy [63]. In people, CT and ultrasound of ICLs is preferred over cytology for the management of ICLs [63]. Based on the results of our study, cytology of aspirates obtained from ICLs cannot be recommended as a method to exclude neoplasia in dogs. Based on the poor sensitivity of cytology for detection of neoplastic cells and the fact that 4 of 13 dogs with histopathology were diagnosed with malignancy, there may be reason for concern with performing renal sparing techniques such as sclerotherapy and deroofing. Conversely, the potential for progression in size of contralateral ICLs with progressive loss of renal function should be carefully considered prior to performing ureteronephrectomy on the most severely affected side in dogs with bilateral lesions. An interesting observation from our study is that dogs with histologically confirmed RCs tend to have > 1 ICL in the ipsilateral or contralateral kidney compared with dogs with histologically confirmed neoplasia. This observation requires further investigation and potentially could be used to guide the most appropriate treatment in dogs with ICLs. The identification of renal epithelial cells on cytology is commonly reported in cases of canine RCs but were identified in only a single case in our study [1, 3, 7]. No bacteria were seen cytologically in any dogs in our study, which is similar to what has been reported previously [1, 3, 7]. Furthermore, culture of cystic fluid yielded no bacterial growth in our study, which is in agreement with the majority of previously reported cases in the literature [1–3, 6, 7].

In our study, 3 of 6 ICLs fully resolved at 45, 51 and 61 days, respectively, following deroofing. All 3 were confirmed as histologically benign RCs. In 1 additional dog diagnosed with RC, the lesion was found to have partially resolved at 44 days after deroofing. Whether the RC of this dog would have further reduced in size with longer follow-up is unknown. Deroofing has been described in 2 dogs with RCs in the literature, both of which had RC resolution at 4.5 and 7 months, respectively [1]. One of these cases was treated with laparoscopy similar to 2 dogs in our study. In humans, laparoscopic deroofing is considered the gold standard for management of symptomatic RCs, especially large cysts in young patients and in cases of failed sclerotherapy or PCD [34]. The overall recurrence rate following laparoscopic deroofing in people is 19% and studies have shown that when combined with omentalization the recurrence rate decreases to 0% [34, 64, 65]. The effect of omentalization in ICL resolution rate cannot be extrapolated from the results presented herein due to the low number of cases. A lower rate of RC recurrence has been reported following laparoscopic deroofing in humans compared with single session sclerotherapy, which is in agreement with the results of our study [66–68]. Whether omentalization in addition to deroofing offers an advantage over deroofing alone with drainage into the abdominal cavity where the omentum naturally resides is unknown. It is important to note that in cases with malignant ICLs, deroofing and omentalization may be inappropriate as it may increase the chances of intraperitoneal tumor spread.

Ureteronephrectomy was most commonly performed as an index treatment in our study, with only 1 dog undergoing this procedure as a revision. This is most likely attributable to individual surgeon preference and even though none of the ICLs included in this study demonstrated radiologic evidence of malignancy, it is possible that a renal sparing technique was considered inappropriate by the attending clinician. This procedure has also been described in 2 dogs with RCs in the veterinary literature but is usually reserved for unilateral renal tumors without evidence of metastasis or contralateral renal impairment [4, 5, 69]. Of those that underwent ureteronephrectomy, 3 were diagnosed with RCs on histopathology, and 4 with neoplasia, 3 malignant and 1 benign. As discussed previously, a concern with performing ureteronephrectomy as index treatment in cases with concurrent smaller ICLs in the contralateral kidney is the potential for progression in size of such ICLs and progressive loss of renal function.

We recognize several important limitations in our study. Due to its retrospective nature, the accuracy of recorded data relies on the completeness of the medical records. The number of cases included is larger than what has been reported previously but remains small. This is a reflection of the infrequent nature of ICLs in dogs. Although a multi-institutional study offered the possibility of gathering a larger number of dogs, such a study design brings with it differences in case management, surgical technique, and follow-up. The study inclusion period extended over 15 years and therefore differences in surgical technique will exist over such a period of time. Due to the non-prospective nature of the study, the selected treatment was not randomized but rather based on individual surgeon preference. The timing of follow-up examinations and imaging studies was not standardized in our study and therefore whether certain ICLs would have eventually resolved with greater follow-up time following a particular treatment cannot be ascertained. Histopathology was not available for 5 dogs in our study. Comparison of the rate of requirement for revision treatment between individual techniques is challenging due to the overall low number of cases within individual treatment categories and the inclusion of neoplastic lesions within certain categories.

## Conclusions

A male sex predilection was found with a 5:1 male:female ratio. Most dogs with ICLs demonstrated nonspecific clinical signs at presentation, with lethargy and decreased appetite being most common. A palpable abdominal mass, tense abdomen during palpation and hypertension were the most common abnormal physical examination findings. A high rate of requirement for revision treatment was identified following PCD. Of the renal-sparing techniques, deroofing +/− omentalization resulted in the highest rate of complete ICL resolution. Almost half of the ICLs were neoplastic, which is particularly important to bear in mind when deciding the most appropriate treatment for affected dogs. While performing ureteronephrectomy in cases with malignancy would be the preferred treatment, obtaining a preoperative diagnosis of malignancy is not always straightforward. Importantly, none of the ICLs included in our study demonstrated clear evidence of malignancy on preoperative abdominal imaging. Cytology of the cystic fluid in our study also failed to diagnose neoplasia in dogs subsequently diagnosed neoplasia on histopathology. Furthermore, performing renal-sparing techniques would be preferred in dogs with bilateral ICLs that could increase in size with time and result in progressive loss of renal function and renal insufficiency. Presence of concurrent ICLs in ipsilateral/contralateral kidney does not appear reliable in differentiating benign from malignant ICLs.

## Methods

Electronic medical records of 11 veterinary academic and private referral institutions were retrospectively reviewed to identify client-owned dogs that underwent PCD, sclerotherapy, surgical deroofing +/− omentalization, or ureteronephrectomy for management of unilateral or bilateral ICLs from January 1^st^ 2004 through July 31^st^ 2021. A minimum of 6 weeks of post- procedural/operative follow-up was required after the last treatment was performed. Details of cases meeting the study inclusion criteria were extracted by contributing surgeons and entered into a dedicated Microsoft Excel spreadsheet.^1^ Exclusion criteria included cases of suspected PKD, PNPs or severe hydronephrosis based on abdominal imaging. ICLs were defined as homogenous anechoic to hypoechoic fluid-filled lesions within the renal parenchyma. Cases suspected to represent malignancy based on preoperative imaging (e.g., presence of a soft tissue mass with accompanying hydronephrosis) were not included in this study. Intrarenal cystic lesions based on abdominal imaging that were subsequently found to be malignant on definitive histopathology were not excluded.

Information extracted from the medical record of dogs meeting inclusion criteria included breed, age, sex (and neuter status) and bodyweight at the time of presentation to the contributing institution; concurrent/historical comorbidities at presentation; medications at presentation; reason for presentation; clinical signs at presentation and duration thereof; abnormal physical examination findings; method (Doppler or oscillometric) and results of blood pressure measurement; and results of preoperative diagnostic tests including complete blood count, biochemical analysis, urinalysis, urine culture, and diagnostic imaging findings (abdominal and thoracic radiography, abdominal ultrasound, computed tomography, and/or intravenous/excretory urogram). For dogs that underwent PCD only, procedure date and occurrence of any periprocedural complications were recorded. For dogs that underwent sclerotherapy, procedure date, sclerosing agent, volume, and concentration, duration of each treatment, and occurrence of any periprocedural complications were recorded. For dogs that underwent surgery, procedure date, procedure type (surgical deroofing/partial nephrectomy/ICL fenestration +/− omentalization, ureteronephrectomy), and pertinent surgical details were recorded. Details of clinicopathologic or microbiologic testing of fluid obtained from ICLs, either preoperatively or intraoperatively, were recorded. Results of histopathologic analysis of excised tissue specimens were also recorded. Treatments performed were divided into index and revision treatments. Index treatment was defined as the first treatment applied to the ICL, whereas revision treatment was defined as any treatment that was subsequently applied to the ICL. Complications were divided into intra-procedural/operative and post-procedural/operative. Intra-procedural/operative complications were defined as any unexpected deviation from the expected course of the procedure/operation. Post-procedural/operative complications were defined as any deviation from the normal post-procedural/operative course. Complications were considered minor if they were self-limiting or resolved with medical treatment alone. Major complications were defined as those requiring additional procedural/surgical intervention or those that resulted in euthanasia or death. Patient outcome was also recorded, including dates of any follow-up examinations; details of follow-up post-procedural/operative abnormal examination findings; persistent clinical signs; results of complete blood count, biochemistry, and urinalysis; and details of any follow-up imaging performed at the contributing institution. Contributing institutions were requested to contact the referring veterinarian or the owners of cases to identify any clinical signs related to ICLs/kidney disease at the time of last recorded alive or dead and cause of death if applicable. Survival time was calculated from the date of index surgery/procedure to the date of last recorded alive, lost-to-follow-up, or death. Death was recorded as renal-disease related, non-renal-disease related or unknown.

Continuous data were tested for normality using the Shapiro-Wilk test. Normally and non-normally distributed continuous data are presented as mean and standard deviation and median and range, respectively. Categorical data are presented as frequency and percentages. All analyses were performed using commercially available software.^2^

## Data Availability

All data generated or analyzed during this study are included in this published article. Raw study data are available for review if required.

## Abbreviations

ICL(s): Intrarenal cystic lesion(s)
PCD: Percutaneous cyst drainage
PKD: Polycystic kidney disease
PNP: Perinephric pseudocyst
PUP: Paraureteral pseudocyst
RC(s): Renal cyst(s)

## Footnotes

1 Microsoft Excel®, Microsoft 365, Microsoft Corporation, WA.

2 IBM SPSS Statistics for Windows, Version 27.0, IBM Corp, Armonk, NY.
